# Preparation of K_2_SbPO_6_-Loaded Porous Geopolymer Particles for Efficient Sr(II) Removal: Adsorption Performance and Mechanism

**DOI:** 10.3390/ma19112319

**Published:** 2026-05-31

**Authors:** Chufeng Cheng, Wei Fang, Gaoshang Ouyang, Jingsong Wang

**Affiliations:** School of Civil Engineering, University of South China, Hengyang 421001, China; 18671965380@163.com (C.C.); fang09114913@163.com (W.F.); oygs@usc.edu.cn (G.O.)

**Keywords:** porous geopolymer, potassium phosphatoantimonate, strontium(II), ion exchange, competing ions

## Abstract

To achieve efficient separation of Sr^2+^ under complex ionic-strength conditions, porous geopolymer particles (PGs) were used as a support to construct a K_2_SbPO_6_-loaded porous geopolymer composite, denoted as K_2_SbPO_6_@PGs, via in situ loading of one-dimensional K_2_SbPO_6_ by a high-temperature solid-state route. Its adsorption performance and mechanism were systematically compared with those of pristine PGs. Structural characterization (SEM/EDS, XRD, FTIR, XPS, and BET) confirmed that the K_2_SbPO_6_ crystalline phase was uniformly anchored onto the PGs framework while preserving interconnected mesoporous channels. K_2_SbPO_6_@PGs exhibited excellent Sr^2+^ removal over a wide pH range (3–12), with a removal efficiency of approximately 92% at pH 3, which was significantly higher than that of PGs (approximately 5%). The isotherm data were better fitted by the Sips model (R^2^ = 0.982), and the maximum adsorption capacity reached 189.35 mg·g^−1^ (theoretical qm = 201.14 mg·g^−1^). Kinetic fitting showed that PGs followed the pseudo-first-order model, whereas K_2_SbPO_6_@PGs were better described by the pseudo-second-order model, indicating that chemical adsorption dominated the process through K^+^/Sr^2+^ exchange and surface complexation. Coexisting-ion experiments demonstrated strong resistance to monovalent ions, whereas Ca^2+^ and Mg^2+^ caused more pronounced competitive effects. The results indicate that PGs mainly provide interconnected mass-transfer pathways and granular structural support, whereas K_2_SbPO_6_ provides selective exchange sites with high affinity for Sr^2+^. The synergy between these two components endows the composite with good pH adaptability and enhanced adsorption performance and suggests its potential for subsequent continuous-flow separation studies.

## 1. Introduction

Nuclear energy is an important low-carbon energy source with high energy density; however, high-level liquid waste (HLLW) generated during spent fuel reprocessing usually contains high acidity, high radioactivity, and complex ionic compositions, making radionuclide separation a key challenge in the nuclear fuel cycle [1,2]. Among the major fission products, ^90^Sr is of particular concern because Sr^2+^ is highly mobile in aqueous systems and exhibits biological behavior similar to Ca^2+^, which may lead to accumulation in bones and teeth and cause long-term radiological risks [3,4]. Therefore, developing efficient adsorbents for Sr^2+^ removal from acidic and complex aqueous systems is of great significance.

Current technologies for Sr^2+^ removal mainly include chemical precipitation/coprecipitation, membrane separation, solvent extraction, adsorption, and ion exchange [1,2,3]. Recent reviews have indicated that adsorption/ion exchange is one of the most promising routes because of its simple operation, low energy consumption, material tunability, and suitability for treating large volumes of dilute wastewater [1,2]. Nevertheless, excess H^+^ and competing ions such as Na^+^, K^+^, Ca^2+^, and Mg^2+^ in complex systems can significantly reduce the adsorption capacity and selectivity of conventional adsorbents [3,4,5,6]. Therefore, adsorbents with high selectivity, acid resistance, and good reusability are still urgently needed.

Inorganic ion-exchange materials have attracted increasing attention for Sr^2+^ capture because of their structural stability and defined exchange sites. Recent studies have reported phosphate-, zeolite-, and polyoxoniobate-based materials for selective Sr^2+^ adsorption, confirming that exchangeable cation sites and oxygen-rich coordination environments are important for Sr^2+^ fixation [4,5,6]. Among acid-resistant inorganic adsorbents, antimony-based materials are especially attractive. Hu et al. developed doped antimony oxides for Sr(II) removal from strongly acidic or simulated HLLW systems, demonstrating the potential of antimony-based frameworks in acidic Sr^2+^ separation [7,8]. More importantly, Guo et al. reported one-dimensional K_2_SbPO_6_ with high Sr^2+^ adsorption capacity and selectivity through K^+^/Sr^2+^ ion exchange [9]. Li et al. further prepared K_2_SbPO_6_/polyacrylonitrile microspheres and showed that shaping powdered K_2_SbPO_6_ improved its operational applicability [10]. However, powdered K_2_SbPO_6_ still faces limitations in solid–liquid separation, column packing, and continuous operation.

Loading highly selective active phases onto porous and formable supports is an effective strategy to improve adsorbent operability. Geopolymers are aluminosilicate-based inorganic materials with good formability, chemical stability, low cost, and tunable porous structures [11,12]. Recent reviews have shown that geopolymers are promising materials for adsorption, stabilization/solidification, and environmental remediation because of their tunable frameworks and surface chemistry [13,14,15]. More recently, shaped porous geopolymers have been developed to improve mass transfer and operational applicability. For example, Ma et al. fabricated hierarchically porous geopolymers for radionuclide adsorption [16], while Wei et al. and Yi et al. prepared geopolymer microspheres for wastewater treatment, demonstrating the advantages of particle-shaped geopolymer adsorbents in adsorption and recovery processes [17,18]. In particular, hierarchically porous zeolite–geopolymer composites have been used for Sr^2+^ decontamination in fixed-bed processes, demonstrating the importance of pore structure and granular stability for continuous adsorption [19,20,21]. However, the intrinsic Sr^2+^ selectivity of porous geopolymers remains limited compared with highly selective ion-exchange phases.

Based on these considerations, K_2_SbPO_6_ was in situ loaded onto porous geopolymer particles to construct K_2_SbPO_6_-loaded porous geopolymer particles, denoted as K_2_SbPO_6_@PGs. This design aims to integrate the selective K^+^/Sr^2+^ exchange ability of K_2_SbPO_6_ with the porous mass-transfer framework and the shaping advantages of PGs. The structures of PGs and K_2_SbPO_6_@PGs were characterized by SEM/EDS, XRD, FTIR, BET, and XPS, and their Sr^2+^ adsorption behavior was systematically compared through pH, dosage, kinetic, isotherm, coexisting-ion, and regeneration experiments. This study clarifies the structure–performance relationship of the composite and reveals the synergistic mechanism between the PG framework and the K_2_SbPO_6_ active phase.

The novelty of this work is not only the use of PGs as a porous support, but also the functional transformation of PGs from a low-selectivity geopolymer adsorbent into a selective Sr(II)-capture composite through the introduction of K_2_SbPO_6_. Pristine PGs possess interconnected pores, good particle formability, and mass-transfer channels, but their Sr(II) adsorption mainly relies on nonspecific framework sites and is strongly limited under acidic and competitive ionic conditions. In contrast, the introduced K_2_SbPO_6_ phase provides specific K^+^/Sr^2+^ exchange sites and oxygen-rich coordination environments, which significantly enhance the Sr(II) affinity and pH adaptability of the composite.

## 2. Materials and Methods

### 2.1. Materials

Metakaolin (MK) was purchased from Shanxi Chaopai Calcined Kaolin Co., Ltd. (Shuozhou, China). K_2_HPO_4_·3H_2_O (99.5 wt%), Sb_2_O_5_ (99 wt%), and KCl (99.99 wt%) were purchased from Shanghai Macklin Biochemical Co., Ltd. (Shanghai, China). The sodium silicate solution was supplied by Zhejiang Jiaxing Yourui Materials and Chemicals Co., Ltd. (Jiaxing, China), with a modulus of 2.3 and a concentration of 43.75%. The mass fractions of SiO_2_ and Na_2_O in the solution were 29.99% and 13.75%, respectively. Sodium dodecyl sulfate (SDS) was purchased from Shanghai Macklin Biochemical Co., Ltd. (Shanghai, China). H_2_O_2_ (30%) was purchased from Hengyang Kaixin Chemical Reagent Co., Ltd. (Hengyang, China). NaCl, KCl, MgCl_2_, and CaCl_2_ (analytical grade) were purchased from Shanghai Macklin Biochemical Co., Ltd. (Shanghai, China). NaOH and HCl (analytical grade) used for pH adjustment were purchased from Hunan Huihong Reagent Co., Ltd. (Changsha, China). Unless otherwise specified, All chemicals were of analytical grade and used without further purification. Deionized water was used throughout the material preparation and the entire experimental study. The chemical composition of MK is summarized in Table 1.

Because radioactive strontium has the same chemical reactivity as its non-radioactive isotopes, natural strontium isotope ^88^Sr is commonly used instead of radioactive ^90^Sr in adsorption experiments for safety and economic reasons. Therefore, ^88^Sr(NO_3_)_2_ (analytical grade, Shanghai Aladdin Biochemical Technology Co., Ltd. (Shanghai, China)) was used in this work.

### 2.2. Synthesis of PGs and K_2_SbPO_6_@PGs

#### 2.2.1. Preparation of PGs

**Figure 1:**
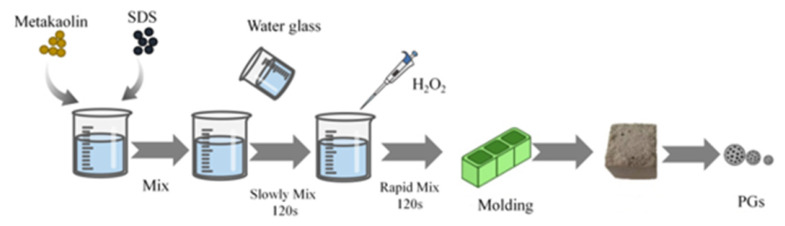
Schematic illustration of the synthesis process of PGs.

For the preparation of porous geopolymer particles, MK (50 g) and SDS (0.35 g) were first thoroughly mixed under stirring, followed by the addition of sodium silicate solution and gentle mixing for 2 min. Then, 0.5 mL of H_2_O_2_ solution was added, and the mixture was stirred rapidly for 2 min to obtain a homogeneous slurry. The freshly prepared slurry was quickly poured into a mold and kept at room temperature for 24 h to complete foaming and stabilize the pore structure. The solidified body was then removed from the mold and transferred to a curing chamber, where it was mildly heat-cured at 60 °C for 24 h to obtain a porous geopolymer. The porous geopolymer was crushed and sieved to collect particles with a diameter of 0.315 mm, denoted as porous geopolymers (PGs). The samples were washed three times with deionized water and dried. The overall synthesis process is schematically illustrated in Figure 1.

#### 2.2.2. Preparation of K_2_SbPO_6_@PGs

**Figure 2:**
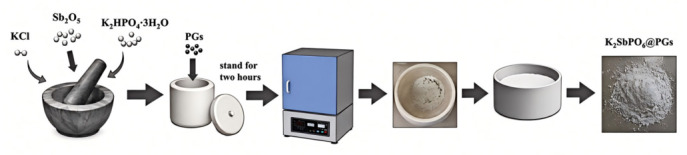
Schematic illustration of the synthesis process of K_2_SbPO_6_@PGs.

K_2_SbPO_6_@PGs was prepared by a high-temperature solid-state route. K_2_HPO_4_·3H_2_O (22.817 g), Sb_2_O_5_ (16.176 g), and KCl (0.743 g) were thoroughly ground to obtain a homogeneous precursor mixture, after which PGs (8.097 g) were added and mixed uniformly. The mixture was transferred to a quartz crucible and allowed to stand for 2 h, preheated at 200 °C for 4 h, heated to 900 °C within 2 h, and maintained at 900 °C for 12 h. The sample was then cooled to 300 °C within 2 h and finally cooled naturally to room temperature to obtain a potassium phosphatoantimonate–porous geopolymer bulk product. The bulk was crushed and sieved to collect particles with a diameter of 0.315 mm, denoted as K_2_SbPO_6_@PGs. The samples were washed three times with deionized water and dried. The overall preparation procedure is shown in Figure 2.

### 2.3. Characterization Methods

#### 2.3.1. SEM and EDS Analysis

The morphology and microstructure of the materials were observed using field-emission scanning electron microscopy (SEM, Sigma 300, Carl Zeiss Microscopy GmbH, Oberkochen, Germany). The powdered samples were evenly spread on conductive tape and, when necessary, sputter-coated with Au/Pt to reduce charging effects. The accelerating voltage was set at 3–10 kV depending on the conductivity of the sample, and secondary electron images were collected for surface morphology analysis.

The elemental composition and spatial distribution were analyzed by energy-dispersive X-ray spectroscopy (EDS, Xplore 30, Oxford Instruments, High Wycombe, UK). Point analysis, line scanning, and elemental mapping were performed to verify the loading of K_2_SbPO_6_ on PGs and the enrichment of Sr after adsorption. The EDS results were used as semiquantitative evidence.

#### 2.3.2. N_2_ Adsorption–Desorption Analysis

The specific surface area and pore structure of the samples were characterized by N_2_ adsorption–desorption measurements using a surface area and porosity analyzer (ASAP 2460, Micromeritics Instrument Corporation, Norcross, GA, USA). Before measurement, the samples were degassed under vacuum at 120 °C for at least 12 h.

The specific surface area was calculated using the Brunauer–Emmett–Teller (BET) method in the relative pressure range of P/P_0_ = 0.05–0.30. The pore size distribution was obtained using the Barrett–Joyner–Halenda (BJH) method based on the desorption branch, and the total pore volume was determined from the adsorption amount at P/P_0_≈ 0.99.

#### 2.3.3. FTIR Analysis

Functional groups and framework bonding information were analyzed by Fourier-transform infrared spectroscopy (FTIR, Nicolet iS20, Thermo Fisher Scientific, Waltham, MA, USA). The spectra were recorded in the wavenumber range of 4000–400 cm^−1^ with a resolution of 4 cm^−1^, and at least 32 scans were collected for each sample.

#### 2.3.4. XRD Analysis

The crystalline phases of the samples were identified by X-ray diffraction (XRD, SmartLab SE, Rigaku Corporation, Tokyo, Japan) using Cu Kα radiation (λ = 1.5406 Å). The scanning range was 5–80° (2θ), with a step size of 0.02°.

The XRD patterns were used to evaluate the amorphous nature of PGs and the formation and stability of the K_2_SbPO_6_ crystalline phase in the composite. Phase identification was performed by comparison with standard PDF cards.

#### 2.3.5. XPS Analysis

The surface chemical states and elemental compositions of the samples before and after Sr(II) adsorption were examined by X-ray photoelectron spectroscopy (XPS, K-Alpha, Thermo Fisher Scientific, Waltham, MA, USA). A monochromatic Al Kα X-ray source was used, and the analysis was conducted under a vacuum lower than 10^−8^ mbar.

The binding energies were calibrated using the C 1s peak at 284.8 eV as a reference. High-resolution spectra of key elements, including O 1s, Si 2p, Al 2p, P 2p, Sb 3d, and Sr 3d, were fitted to clarify the adsorption mechanisms, such as ion exchange and surface complexation.

### 2.4. Adsorption and Desorption Experiments

Sr(II) stock solution was prepared by dissolving 2.415 g of Sr(NO_3_)_2_ in 1000 mL of deionized water and then diluted to the desired working concentrations before use. Unless otherwise specified, batch adsorption experiments were conducted by adding 10 mg of PGs or K_2_SbPO_6_@PGs into 10 mL of Sr(II) solution in Erlenmeyer flasks, followed by shaking at 150 rpm and 25 °C. After adsorption, the suspension was filtered through a 0.22 μm syringe filter, and the filtrate was diluted with 2% HNO_3_ for Sr(II) determination by atomic absorption spectrometry (AA-6300, Shimadzu Corporation, Kyoto, Japan). The specific experimental conditions for pH-effect, dosage-effect, adsorption kinetics, adsorption isotherm, coexisting-ion, and regeneration experiments are described below.

For pH-effect experiments, 10 mg of adsorbent was added to 10 mL of Sr(II) solution, and the initial solution pH was adjusted to 3.04–12.02 using 0.01–0.1 M HCl or 0.01–0.1 M NaOH before adsorption. For dosage-effect experiments, 5–50 mg of PGs or K_2_SbPO_6_@PGs was added to 50 mL of Sr(II) solution, corresponding to an adsorbent dosage of 0.1–1.0 g·L^−1^. The solution volume and other experimental conditions were kept constant to evaluate the effect of adsorbent dosage on Sr(II) removal efficiency and adsorption capacity. For adsorption kinetic experiments, 10 mg of PGs or K_2_SbPO_6_@PGs was added to 10 mL of 100 mg·L^−1^ Sr(II) solution, and samples were collected at predetermined time intervals. For adsorption isotherm experiments, 10 mg of adsorbent was added to 10 mL of Sr(II) solutions with initial concentrations ranging from 49.09 to 950.71 mg·L^−1^, and the equilibrium data were fitted using isotherm models. For coexisting-ion experiments, 10 mg of adsorbent was added to 10 mL of Sr(II) solution containing Na^+^, K^+^, Mg^2+^, or Ca^2+^ at different mass ratios relative to Sr^2+^, with Sr^2+^ concentrations ranging from 94.26 to 98.76 mg·L^−1^ and the solution pH adjusted to 6.06–8.17.

(1) $$q=\frac{\left(C_{0}-C_{t}\right)\times V}{m_{0}}$$

where *q* is the adsorption capacity of the adsorbent toward Sr^2+^ (mg·g^−1^); *C*_0_ is the initial concentration of Sr^2+^ (mg·L^−1^); *C_t_* is the Sr^2+^ concentration at contact time t (mg·L^−1^); *V* is the solution volume (mL); and *m*_0_ is the adsorbent dosage (mg).

(2) $$R=\frac{C_{0}-C_{t}}{C_{0}}\times 100\%$$

where *R* is the removal efficiency (%); *C*_0_ is the initial concentration of Sr^2+^ (mg·L^−1^); and *C_t_* is the Sr^2+^ concentration at contact time *t* (mg·L^−1^).

After adsorption, the K_2_SbPO_6_@PGs were separated by quantitative filter paper, placed in 5 mL of 2 mol·L^−1^ KCl solution, and shaken at 150 rpm and 25 °C for 8 h in a thermostatic shaker. The adsorbent was then filtered, washed, and dried for subsequent regeneration experiments.

For consecutive regeneration and saturation experiments, one adsorption cycle was first conducted under the above static adsorption conditions. The Sr(II)-loaded K_2_SbPO_6_@PGs were separated by quantitative filter paper, washed gently with deionized water, and then placed in 5 mL of KCl solution (1.0, 1.5, or 2.0 mol·L^−1^) as the eluent. The mixture was shaken at 150 rpm and 25 °C for 8 h. After elution, the adsorbent was filtered, washed, dried, and reused for the next adsorption cycle. Five consecutive adsorption–regeneration cycles were performed, and the adsorption capacity after each cycle was used to evaluate regeneration performance.

All batch adsorption experiments were independently performed in triplicate under identical conditions. The adsorption capacity and removal efficiency are reported as the mean values of three parallel measurements, and the error bars in the corresponding figures represent the standard deviation. When the error bars are not visible, the standard deviation is smaller than the symbol size. Model fitting was carried out using Origin 2024 software (Version 10.1.0.178, OriginLab Corporation, Northampton, MA, USA), and the coefficient of determination (R^2^) was used to evaluate the fitting quality.

## 3. Results and Discussion

### 3.1. Structural and Surface Characterization of PGs and K_2_SbPO_6_@PGs

#### 3.1.1. Morphology and Elemental Distribution

**Figure 3:**
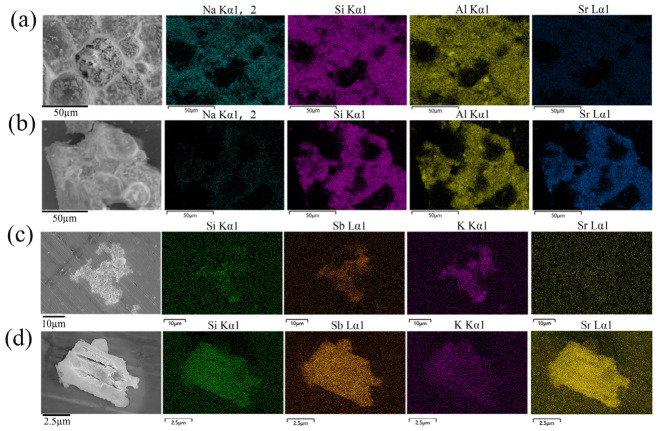
Elemental mapping images of the samples before and after Sr(II) adsorption: (**a**) PGs before adsorption; (**b**) PGs after adsorption; (**c**) K_2_SbPO_6_@PGs before adsorption; and (**d**) K_2_SbPO_6_@PGs after adsorption. In the EDS elemental mapping images of PGs, cyan, purple, yellow, and blue represent the distributions of Na, Si, Al, and Sr, respectively. In the EDS elemental mapping images of K_2_SbPO_6_@PGs, green, orange, purple, and yellow represent the distributions of Si, Sb, K, and Sr, respectively.

As shown in Figure 3a,b, PGs exhibited an irregular porous morphology, and Na, Si, and Al were distributed throughout the particle region, indicating the formation of an aluminosilicate geopolymer framework. After Sr(II) adsorption, a Sr signal appeared on the PGs surface, confirming that Sr(II) was retained by PGs. However, the Sr distribution was relatively dispersed, suggesting limited Sr(II) uptake by pristine PGs.

For K_2_SbPO_6_@PGs, Sb and K were clearly observed in the composite particles before adsorption, confirming the successful introduction of K_2_SbPO_6_-related components into the PGs matrix (Figure 3c). After Sr(II) adsorption, an evident Sr signal appeared in K_2_SbPO_6_@PGs and showed more pronounced enrichment than that in PGs (Figure 3d), indicating the stronger Sr(II) uptake ability of the composite.

**Figure 4:**
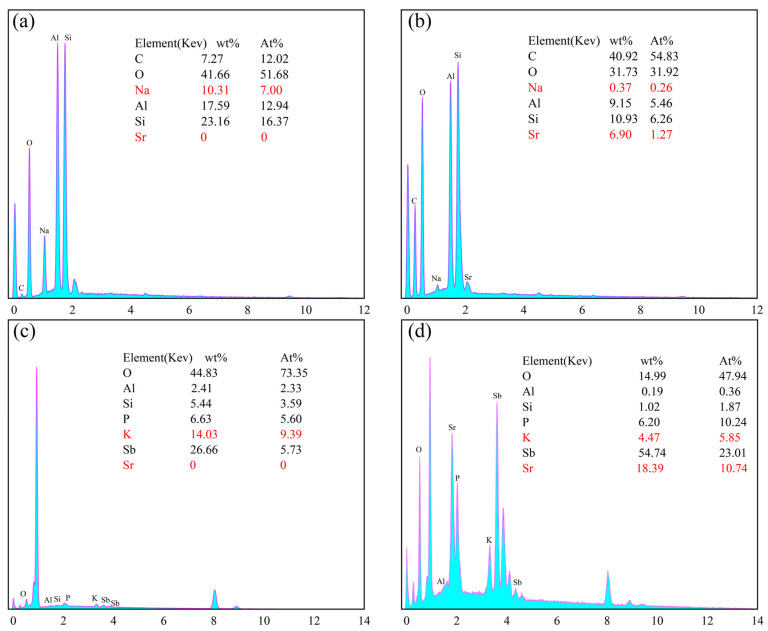
EDS spectra of PGs and K_2_SbPO_6_@PGs before and after Sr(II) adsorption: (**a**) PGs before adsorption; (**b**) PGs after adsorption; (**c**) K_2_SbPO_6_@PGs before adsorption; (**d**) K_2_SbPO_6_@PGs after adsorption.

The EDS spectra in Figure 4 further confirm the elemental composition changes before and after adsorption. For PGs, O, Na, Al, and Si were detected before adsorption, while a Sr signal appeared after adsorption and the Na content decreased from 10.31 wt.% to 0.37 wt.% (Figure 4a,b). For K_2_SbPO_6_@PGs, O, Al, Si, P, K, and Sb were detected before adsorption, confirming the presence of K_2_SbPO_6_-related components (Figure 4c). After adsorption, the Sr content reached 18.39 wt.%, while the K content decreased from 14.03 wt.% to 4.47 wt.% (Figure 4d). These results provide direct elemental evidence for the higher Sr(II) uptake of K_2_SbPO_6_@PGs than PGs. The detailed adsorption mechanism is further discussed in Section 3.7.

The C signal observed in the EDS spectra is mainly attributed to adventitious carbon on the sample surface and/or conductive carbon tape used during SEM/EDS sample preparation, rather than to the raw materials or the formation of a carbon-containing active phase.

#### 3.1.2. Crystalline Phase Composition

**Figure 5:**
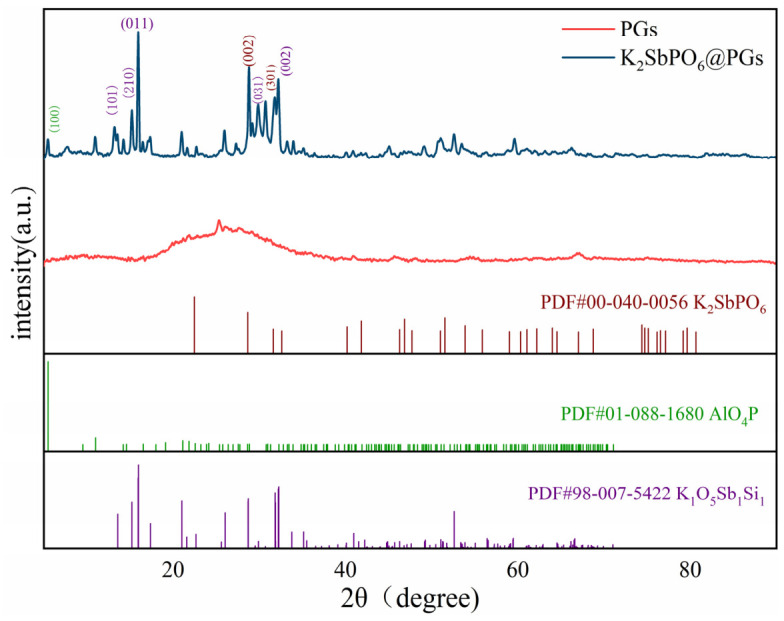
XRD patterns of PGs and K_2_SbPO_6_@PGs and the corresponding phase identification results.

XRD was used to analyze the phase compositions of PGs and K_2_SbPO_6_@PGs, as shown in Figure 5. The diffraction pattern of PGs mainly exhibited a broad diffuse hump without obvious sharp crystalline peaks, indicating that the main structure was an amorphous aluminosilicate framework, which is a typical feature of geopolymer materials. This result confirms that the foaming and curing process produced a porous framework dominated by an amorphous phase. In contrast, K_2_SbPO_6_@PGs displayed several clear crystalline diffraction peaks, demonstrating the formation of new crystalline phases after high-temperature solid-state compositing. According to the phase-indexing results labeled in Figure 5, these diffraction peaks can be assigned to K_2_SbPO_6_ (PDF#00-040-0056), AlO_4_P (PDF#01-088-1680), and K_1_O_5_Sb_1_Si_1_ (PDF#98-007-5422). The appearance of characteristic K_2_SbPO_6_ peaks confirms that the target potassium antimony phosphate active phase was successfully constructed on the PG support. Meanwhile, the peaks associated with AlO_4_P and K_1_O_5_Sb_1_Si_1_ suggest that interfacial reactions may have occurred between the precursors and the geopolymer aluminosilicate matrix during the high-temperature solid-state reaction.

#### 3.1.3. Functional Groups and Framework Bonds

**Figure 6:**
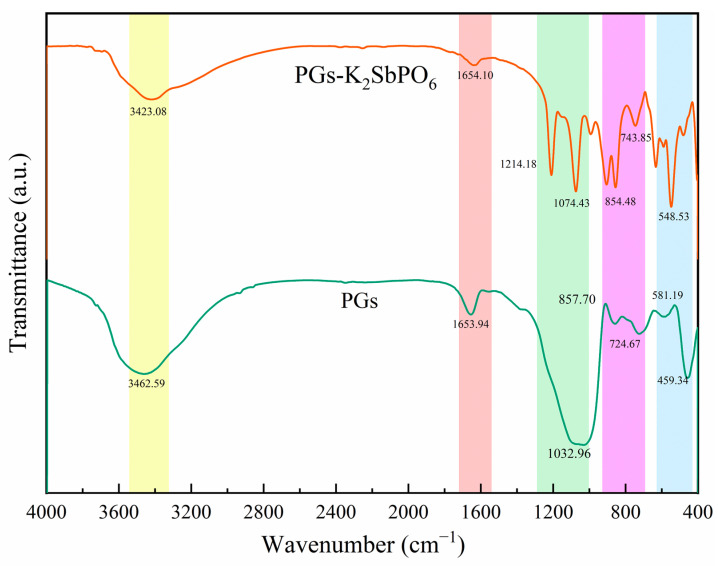
FTIR spectra of PGs and K_2_SbPO_6_@PGs. The colored shaded regions are used to highlight the characteristic absorption bands discussed in the text.

The FTIR spectra of PGs and K_2_SbPO_6_@PGs are shown in Figure 6. Both samples exhibited broad bands at 3423–3463 cm^−1^ and a distinct absorption peak at approximately 1654 cm^−1^, corresponding to the O–H stretching vibration of surface hydroxyl/adsorbed water and the H–O–H bending vibration of adsorbed water, respectively, indicating the presence of abundant oxygen-containing groups and hydrophilic environments on the material surfaces, which are favorable for ion diffusion and interfacial interactions. For PGs, the main absorption band at 1032.96 cm^−1^ can be assigned to the asymmetric stretching vibration of Si–O–T (T = Si or Al) in the aluminosilicate framework, which is a typical feature of geopolymer gel structures. In addition, the absorption bands at 857.70, 724.67, 581.19, and 459.34 cm^−1^ are also associated with framework-related Si–O and Al–O vibrations, indicating that PGs mainly consist of a geopolymeric aluminosilicate network.

By contrast, K_2_SbPO_6_@PGs retained the geopolymer framework bands while showing new or enhanced absorption peaks at 1214.18 and 1074.43 cm^−1^, which can be assigned to the P–O stretching vibration of phosphate groups. Phosphate-related vibrational features were also observed near 854.48, 743.85, and 548.53 cm^−1^. These phosphate-related bands, which appeared or became significantly enhanced only in the composite, further support the successful introduction of K_2_SbPO_6_ active domains into the PGs matrix. Therefore, the FTIR results indicate that K_2_SbPO_6_@PGs retains the aluminosilicate framework of PGs while introducing phosphate-related functional groups. The role of these functional groups in Sr(II) adsorption is further discussed in Section 3.7.

#### 3.1.4. Textural Properties and Pore Structure

**Figure 7:**
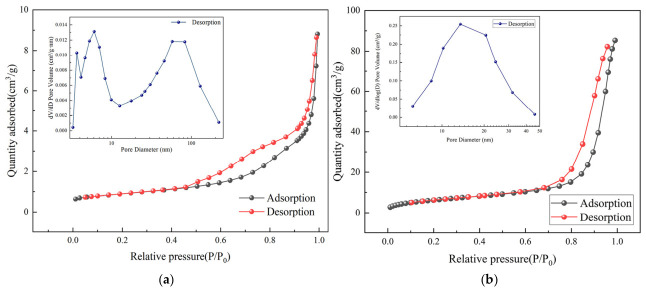
N_2_ adsorption–desorption isotherms and pore size distributions of PGs and K_2_SbPO_6_@PGs: (**a**) K_2_SbPO_6_@PGs and (**b**) PGs.

The pore-structure characteristics of PGs and K_2_SbPO_6_@PGs were analyzed by N_2_ adsorption–desorption measurements, and the results are shown in Figure 7. Both materials exhibited typical type IV adsorption isotherms with obvious hysteresis loops, indicating the presence of mesoporous structures. According to Table 2, PGs had a relatively high specific surface area (23.924 m^2^·g^−1^), pore volume (0.131 cm^3^·g^−1^), and average pore diameter (22.20 nm), indicating that the foaming and curing process successfully constructed an interconnected porous framework that favors rapid ion transport during adsorption.

After loading K_2_SbPO_6_, the specific surface area and pore volume of the composite decreased to 3.144 m^2^·g^−1^ and 0.014 cm^3^·g^−1^, respectively, while the average pore diameter decreased to 13.18 nm. This change may be attributed to the deposition and growth of K_2_SbPO_6_-related crystalline phases on the pore walls or pore entrances, as well as the partial filling or narrowing of local pores during high-temperature treatment. Nevertheless, K_2_SbPO_6_@PGs still retained a type IV isotherm and a distinct hysteresis loop, indicating that its mesoporous transport channels were not completely lost and that the basic porous framework of PGs was largely preserved during composite formation. Although the specific surface area and pore volume decreased after K_2_SbPO_6_ loading, the composite retained mesoporous transport channels. These textural features suggest that PGs can still provide a porous structural framework for solution–solid contact. The relationship between the pore structure and Sr(II) adsorption performance is further discussed together with the adsorption results in the following sections.

#### 3.1.5. Surface Chemical States and Adsorption Evidence

**Figure 8:**
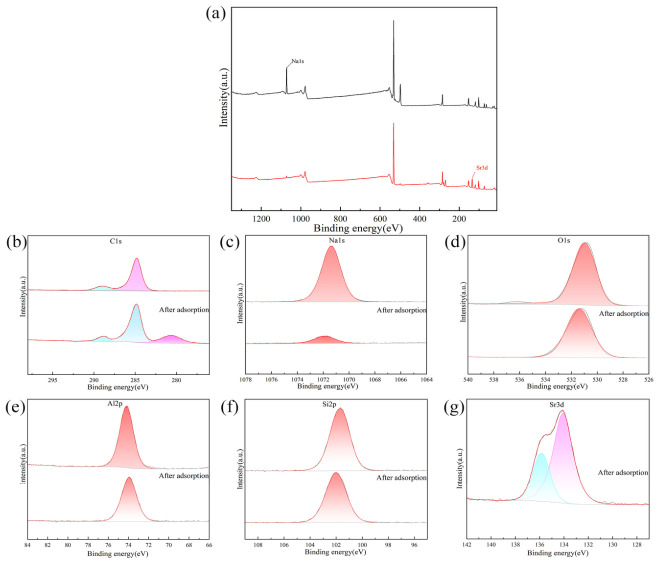
XPS spectra of PGs before and after Sr(II) adsorption: (**a**) survey spectra and (**b**–**g**) high-resolution spectra and peak fitting of C 1s, Na 1s, O 1s, Al 2p, Si 2p, and Sr 3d. The colored shaded areas are used only to distinguish different fitted peaks; the grey dashed lines represent the baselines, the grey jagged lines represent the experimental data, and the red curves represent the fitted curves.

**Figure 9:**
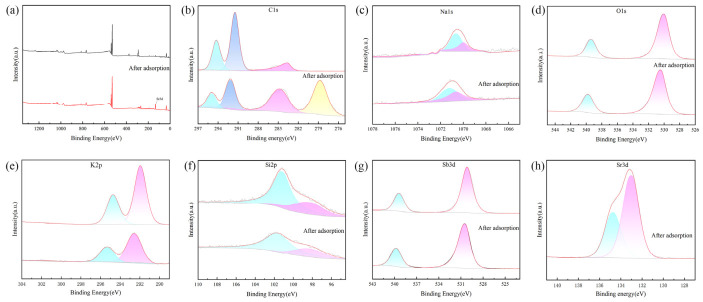
XPS spectra of K_2_SbPO_6_@PGs before and after Sr(II) adsorption: (**a**) survey spectra and (**b**–**h**) high-resolution spectra and peak fitting of C 1s, Na 1s, O 1s, K 2p, Si 2p, Sb 3d, and Sr 3d. The colored shaded areas are used only to distinguish different fitted peaks; the grey dashed lines represent the baselines, the grey jagged lines represent the experimental data, and the red curves represent the fitted curves.

To further clarify the surface chemical states and adsorption mechanism, XPS analyses of PGs and K_2_SbPO_6_@PGs before and after Sr(II) adsorption were carried out, and the results are shown in Figure 8 and Figure 9. Sr-related signals appeared in both materials after adsorption, indicating that Sr(II) was successfully immobilized on the material surface or near-surface region. At the same time, framework elements such as Si, Al, and O remained present after adsorption, suggesting that the overall host structure was stable during the adsorption process.

For PGs, Na-related signals were clearly detected before adsorption, whereas the appearance of Sr 3d peaks after adsorption, together with the obvious change in the Na1s signal, indicates that Na^+^/Sr^2+^ exchange participated in Sr(II) adsorption. In addition, the change in the O1s spectral profile suggests that oxygen-containing groups in the geopolymer framework also interacted with Sr(II), likely through surface complexation or coordination. For K_2_SbPO_6_@PGs, distinct K2p and Sb3d peaks were observed before adsorption, confirming the successful introduction of the K_2_SbPO_6_-containing active phase. After adsorption, the Sr 3d signal became evident, while the K 2p signal decreased significantly. This change is consistent with the Sr enrichment and K depletion observed by EDS, confirming that K^+^/Sr^2+^ exchange occurred in the composite. Meanwhile, the overall Sb 3d signal changed only slightly, indicating that the Sb–P–O framework remained structurally stable during adsorption. Combined with the changes in O 1s and the FTIR results, it can also be inferred that oxygen-containing sites from phosphate/antimonate domains and the geopolymer matrix contributed to the interfacial stabilization of adsorbed Sr(II). Overall, the XPS results show that Sr(II) removal by PGs mainly involves Na^+^/Sr^2+^ exchange and oxygen-containing sites, whereas K_2_SbPO_6_@PGs exhibits a stronger adsorption mechanism dominated by K^+^/Sr^2+^ exchange, which is consistent with its superior adsorption performance relative to PGs.

### 3.2. Effect of pH on Sr(II) Adsorption by PGs and K_2_SbPO_6_@PGs

**Figure 10:**
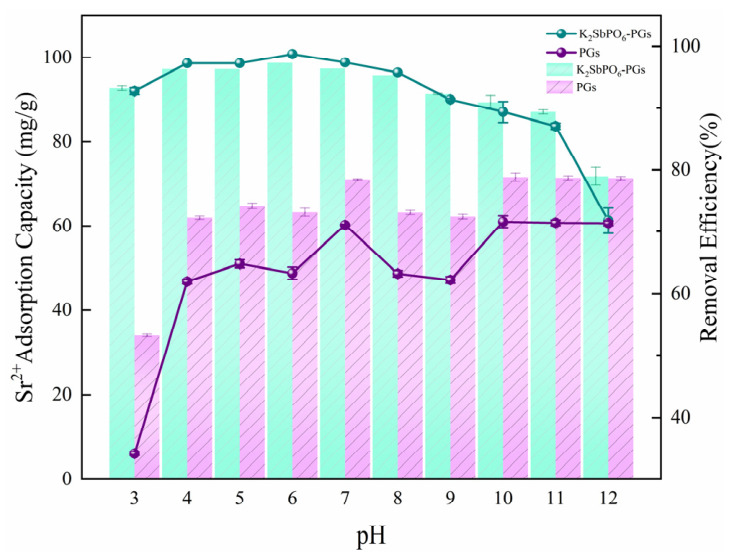
Effect of solution pH on Sr(II) adsorption by PGs and K_2_SbPO_6_@PGs. The columns represent adsorption capacity, and the curves represent removal efficiency.

As shown in Figure 10, the effect of pH on Sr(II) adsorption was markedly different for PGs and K_2_SbPO_6_@PGs. Under acidic conditions, Sr(II) adsorption by PGs was strongly suppressed, mainly due to the competition between excess H^+^ and Sr^2+^ for the limited adsorption/exchange sites. In contrast, K_2_SbPO_6_@PGs maintained high Sr(II) removal efficiency over a wide pH range, indicating that the introduction of K_2_SbPO_6_ significantly improved the acid adaptability and Sr(II) affinity of the composite. This improvement can be mainly attributed to the K_2_SbPO_6_-derived K^+^/Sr^2+^ exchange sites and the cooperative contribution of oxygen-containing groups confirmed by FTIR and XPS analyses. Considering both adsorption capacity and removal efficiency, K_2_SbPO_6_@PGs exhibited a broad suitable pH range of 3–12, with particularly stable performance under acidic to neutral conditions. Therefore, instead of showing a single optimal pH value, K_2_SbPO_6_@PGs demonstrated a wide pH adaptability for Sr(II) removal.

### 3.3. Effect of Adsorbent Dosage

**Figure 11:**
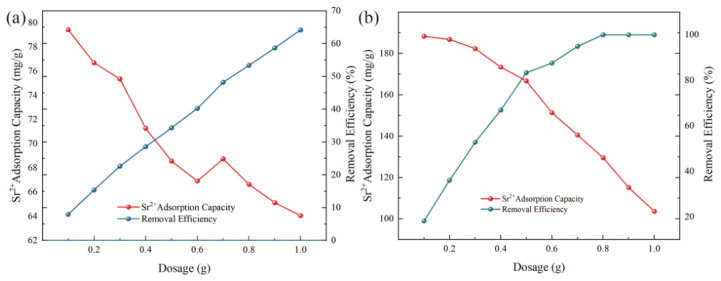
Effect of adsorbent dosage on Sr(II) removal: (**a**) PGs and (**b**) K_2_SbPO_6_@PGs. Relationship between removal efficiency (R) and adsorption capacity(q) as a function of dosage (other conditions are given in Section 2.4).

As shown in Figure 11, the removal efficiency of Sr(II) by both PGs and K_2_SbPO_6_@PGs increased with increasing adsorbent dosage, whereas the adsorption capacity per unit mass decreased accordingly. The increase in removal efficiency is mainly attributed to the increase in the total number of available adsorption/exchange sites, while the decrease in unit adsorption capacity is related to the lower utilization efficiency of active sites under a fixed initial Sr(II) concentration. For K_2_SbPO_6_@PGs, the removal efficiency approached a plateau when the dosage increased to 0.8–1.0 g·L^−1^; therefore, 1.0 g·L^−1^ was selected as a suitable dosage for subsequent static adsorption tests. Throughout the entire dosage range, K_2_SbPO_6_@PGs consistently exhibited higher removal efficiency and adsorption capacity than PGs, indicating that the introduction of K_2_SbPO_6_ significantly enhanced the effective Sr(II) uptake ability of the material.

### 3.4. Adsorption Kinetics

The adsorption processes of Sr^2+^ on PGs and K_2_SbPO_6_@PGs could be fitted by the pseudo-first-order (PFO) and pseudo-second-order (PSO) kinetic models, respectively [22,23]. To elucidate the adsorption mechanism, the experimental data were fitted to kinetic models, and the corresponding parameters were compared.

The linear forms of the two models are as follows:

(3) $$q_{t}=q_{e}\times (1-e^{-k_{1}t})$$

(4) $$q_{t}=\frac{k_{2}q_{e}^{2}t}{1+k_{2}q_{e}t}$$

where *q_t_* and *q_e_* are the adsorption capacities at time *t* (min) and at equilibrium (mg·g^−1^), respectively, and *k*_1_ and *k*_2_ are the pseudo-first-order and pseudo-second-order rate constants, respectively.

**Figure 12:**
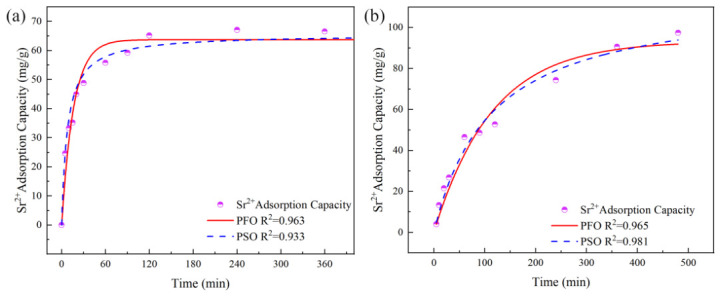
Adsorption kinetics of Sr(II) on (**a**) PGs and (**b**) K_2_SbPO_6_@PGs and the corresponding fitting curves based on the pseudo-first-order (PFO) and pseudo-second-order (PSO) models.

As shown in Figure 12, PGs exhibited a faster Sr^2+^ uptake rate in the initial stage (0–30 min) and approached equilibrium within approximately 60 min, which is usually associated with rapid external diffusion/pore diffusion and the predominance of readily accessible surface sites in its open porous structure. In contrast, K_2_SbPO_6_@PGs adsorbed Sr^2+^ more slowly and reached equilibrium after approximately 480 min, indicating that, in addition to mass transfer, ion exchange and surface chemical interactions contributed more significantly to the adsorption process in the composite. Notably, compared with the longer equilibrium time reported for powdered K_2_SbPO_6_ (approximately 720 min), the composite particles reached equilibrium more rapidly while maintaining high capacity, suggesting that the porous structure of the PGs helped reduce particle aggregation of the crystalline phase and improve the solution–solid interfacial mass transfer.

The fitting results further support this interpretation. PGs showed a higher fitting coefficient for the pseudo-first-order model (R^2^ = 0.963) than for the pseudo-second-order model (R^2^ = 0.933), indicating that its adsorption behavior was more strongly influenced by rapid mass transfer and the accessibility of surface sites. In contrast, K_2_SbPO_6_@PGs exhibited a higher R^2^ value for the pseudo-second-order model (R^2^ = 0.981) than for the pseudo-first-order model (R^2^ = 0.965), indicating that chemical adsorption, including ion exchange and coordination, dominated the rate-controlling step. Combined with the XPS results, the adsorption process of K_2_SbPO_6_@PGs can be reasonably understood as a synergistic process involving rapid initial surface accumulation, followed by slower ion-exchange/interfacial reactions, and finally equilibrium.

### 3.5. Adsorption Isotherms

**Figure 13:**
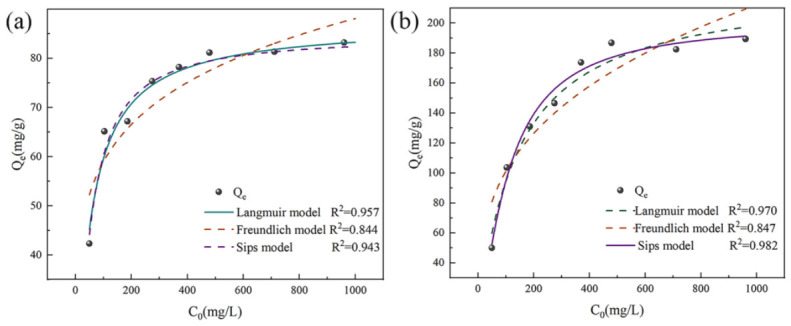
Adsorption isotherms of Sr(II) on (**a**) PGs and (**b**) K_2_SbPO_6_@PGs and the corresponding fitting curves based on the Langmuir, Freundlich, and Sips models.

As shown in Figure 13, the equilibrium adsorption capacities (q_e_) of both PGs and K_2_SbPO_6_@PGs increased with increasing initial Sr^2+^ concentration and gradually approached a plateau in the high-concentration region, indicating a transition from unsaturated adsorption sites to the gradual occupation and eventual saturation of active sites. Therefore, isotherm fitting can be used to quantitatively analyze the maximum adsorption capacity and the energy distribution of adsorption sites, providing key parameters for material optimization and process scale-up.

To investigate the adsorption characteristics and surface-site distributions of the two adsorbents, the experimental data were fitted using the Langmuir, Freundlich, and Sips (Langmuir–Freundlich) models [24,25,26]. The corresponding equations are as follows:

(5) $$\frac{C_{e}}{q_{e}}\;=\;\frac{1}{q_{m}k_{l}}+\frac{C_{e}}{q_{m}}$$

(6) $$Inq_{e}=\frac{1}{n}InC_{e}+Ink_{f}$$

(7) $$q_{e}=\frac{q_{m}{\left(K_{S}C_{e}\right)}^{\beta }}{1+{\left(K_{S}C_{e}\right)}^{\beta }}$$

where *q_e_* and *q_m_* are the equilibrium adsorption capacity (mg·g^−1^) and the maximum monolayer adsorption capacity (mg·g^−1^), respectively; *C_e_* is the equilibrium concentration of Sr^2+^ (mg·L^−1^); *K*_1_, *K_f_*, and *Ks* are the Langmuir, Freundlich, and Sips adsorption constants, respectively; and β is the heterogeneity index (dimensionless).

The fitting results show that the adsorption data of K_2_SbPO_6_@PGs were best described by the Sips model (R^2^ = 0.982), indicating that the composite surface possessed heterogeneous sites while also exhibiting monolayer-like adsorption characteristics. By contrast, the adsorption behavior of PGs was better fitted by the Langmuir model (R^2^ = 0.957), suggesting a relatively homogeneous distribution of accessible sites. The introduction of K_2_SbPO_6_ not only increased the adsorption capacity and affinity but also significantly enhanced the heterogeneity of the adsorption sites in the composite.

According to the Sips model, the theoretical maximum adsorption capacity of K_2_SbPO_6_@PGs was 201.14 mg·g^−1^, which was close to the experimental maximum value of 189.35 mg·g^−1^, indicating that the model could reasonably describe its adsorption behavior. Moreover, this capacity was significantly higher than that of PGs (experimental value: 83.2 mg·g^−1^) and also higher than the maximum adsorption capacity reported for powdered K_2_SbPO_6_ (175.90 mg·g^−1^), suggesting that the synergy between the porous support and the crystalline ion-exchange sites effectively improved the utilization efficiency of active sites and the overall adsorption capacity.

The corresponding isotherm fitting parameters are summarized in Table 3.

### 3.6. Effect of Coexisting Ions

**Figure 14:**
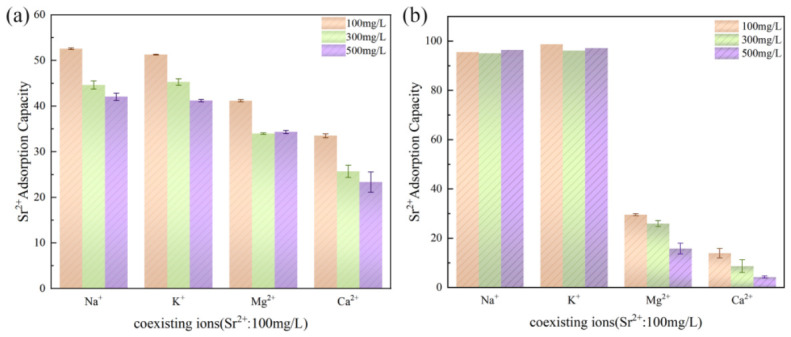
Effect of coexisting ions on Sr(II) adsorption: (**a**) PGs and (**b**) K_2_SbPO_6_@PGs. Comparison of Sr(II) adsorption capacities under different concentrations of Na^+^, K^+^, Mg^2+^, and Ca^2+^.

As shown in Figure 14, coexisting ions (Na^+^, K^+^, Ca^2+^, and Mg^2+^) can reduce the effective adsorption of Sr^2+^ by competing for adsorption/exchange sites and increasing the ionic strength of the solution. Both adsorbents were more sensitive to divalent ions than to monovalent ions.

For K_2_SbPO_6_@PGs, Na^+^ and K^+^ exerted only limited influence on the removal performance, indicating a certain resistance to interference from monovalent ions. However, the removal efficiency decreased markedly in the presence of Ca^2+^ or Mg^2+^, and the inhibition was most pronounced for these divalent ions. This suggests that divalent ions can more readily compete for the exchange channels of K_2_SbPO_6_ or interact more strongly with oxygen-containing groups, thereby displacing Sr^2+^ from its preferred sites. In contrast, PGs showed different degrees of performance deterioration in the presence of both monovalent and divalent ions, reflecting their stronger dependence on nonspecific electrostatic adsorption/physical adsorption. Among these ions, Ca^2+^ and Mg^2+^ again exhibited the strongest inhibition, which can be attributed to their higher charge density and stronger site occupation ability. These results indicate that the selectivity of K_2_SbPO_6_@PGs mainly originates from specific ion-exchange sites, while the stronger competitive effects of Ca^2+^ and Mg^2+^ should be considered under complex ionic-background conditions.

### 3.7. Adsorption Mechanism

Taken together, the adsorption experiments and structural characterizations indicate that the superior Sr^2+^ removal by K_2_SbPO_6_@PGs originates from the coupled contribution of the porous PG support and the K_2_SbPO_6_ active phase. First, the PG framework provides interconnected pore channels and granular structural support, which facilitates solution-solid contact and Sr^2+^ transport to the active interfaces. Second, K_2_SbPO_6_ introduces exchangeable K^+^ sites with high affinity for Sr^2+^, serving as the dominant functional phase for selective uptake. This interpretation is supported by four complementary pieces of evidence: (i) SEM/EDS mapping showed preferential Sr enrichment in Sb/K-containing regions after adsorption; (ii) EDS and XPS both revealed a characteristic Sr enrichment-K attenuation trend, confirming K^+^/Sr^2+^ exchange; (iii) the relatively stable Sb 3d signal indicated that the Sb-P-O framework was largely preserved during adsorption; and (iv) FTIR and O 1s XPS variations suggested that oxygen-containing groups from both the geopolymer framework and phosphatoantimonate domains contributed to interfacial coordination. The better fit of K_2_SbPO_6_@PGs to the pseudo-second-order kinetic model and Sips isotherm further supports an adsorption process governed by chemisorption-related ion exchange and heterogeneous active sites. This evidence chain explains the high capacity, broad pH adaptability, and stronger sensitivity to divalent competing ions observed for K_2_SbPO_6_@PGs.

### 3.8. Cyclic Adsorption Performance After Regeneration

**Figure 15:**
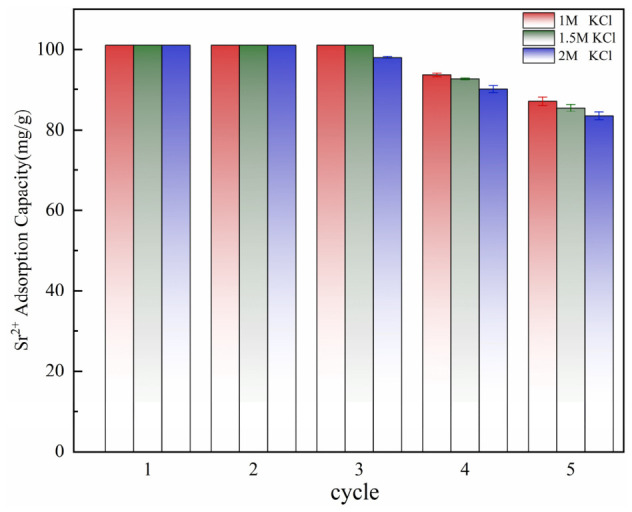
Cyclic Sr(II) adsorption capacity of K_2_SbPO_6_@PGs over five consecutive adsorption–regeneration cycles using 1.0, 1.5, and 2.0 mol·L^−1^ KCl solutions as regenerants.

As shown in Figure 15, the cyclic adsorption capacity of K_2_SbPO_6_@PGs gradually decreased with increasing cycle number after regeneration with KCl solutions. During the first two cycles, the adsorption capacity remained nearly unchanged, indicating that most active sites could be effectively recovered at the initial stage. After five cycles, the adsorption capacities remained at 87.10, 85.46, and 83.43 mg·g^−1^ for 1.0, 1.5, and 2.0 mol·L^−1^ KCl, respectively, corresponding to more than 82% of the initial adsorption capacity. These results indicate that KCl regeneration can partially restore the K^+^/Sr^2+^ exchange sites and that K_2_SbPO_6_@PGs possesses good cyclic adsorption stability.

It should be noted that the regeneration experiment in this work was mainly designed to evaluate the reversibility of Sr(II) adsorption and the cyclic stability of K_2_SbPO_6_@PGs under simulated non-radioactive conditions. For practical radioactive-waste treatment, regeneration is not the only disposal option. The Sr-loaded adsorbent can be directly immobilized or solidified in cementitious or geopolymer-based matrices for safe disposal. If chemical regeneration is adopted, the Sr-containing eluate should be collected as secondary radioactive liquid waste and further concentrated, immobilized, or solidified according to radioactive-waste management requirements, rather than being discharged or returned directly to a nuclear reactor.

### 3.9. Comparison with Reported Sr(II) Adsorbents

To further evaluate the adsorption performance of K_2_SbPO_6_@PGs, representative Sr(II) adsorbents reported in recent studies were compared, as summarized in Table 4.

As summarized in Table 4, K_2_SbPO_6_@PGs exhibits a competitive Sr(II) adsorption performance compared with representative adsorbents reported in recent studies. The experimental maximum adsorption capacity of K_2_SbPO_6_@PGs reached 189.35 mg·g^−1^, which is higher than that of pristine PGs, powdered 1D- K_2_SbPO_6_, K_2_SbPO_6_/PAN composite microspheres, Na_3_FePO_4_CO_3_, layered zirconium phosphate fluoride, and several shaped composite adsorbents. Although some ZrP-based magnetic aerogels and mesoporous hybrid materials show higher capacities, K_2_SbPO_6_@PGs presents a more balanced performance by combining relatively high Sr(II) uptake, an inorganic porous skeleton, granular formability, and a clear K^+^/Sr^2+^ ion-exchange mechanism. In particular, the adsorption capacity increased from 83.2 mg·g^−1^ for PGs to 189.35 mg·g^−1^ after K_2_SbPO_6_ loading, despite the decrease in BET surface area, indicating that the enhanced performance mainly originates from the introduction of selective K_2_SbPO_6_ active domains rather than a surface-area effect. Therefore, K_2_SbPO_6_@PGs demonstrates the advantage of transforming low-selectivity PGs into a selective Sr(II)-capture composite while retaining the structural and mass-transfer benefits of the geopolymer support.

## 4. Conclusions

K_2_SbPO_6_@PGs composite particles were successfully prepared by in situ loading of K_2_SbPO_6_ onto porous geopolymer particles through a high-temperature solid-state route. Structural characterizations confirmed that K_2_SbPO_6_-related crystalline phases were introduced into the PG framework while the composite retained mesoporous transport channels. SEM/EDS and XPS results indicated Sr enrichment and K attenuation after adsorption, confirming that K^+^/Sr^2+^ ion exchange is a key mechanism for Sr(II) capture.

Compared with pristine PGs, K_2_SbPO_6_@PGs exhibited much better Sr(II) adsorption performance over a wide pH range. The Sips model best described the isotherm data of K_2_SbPO_6_@PGs, giving a theoretical maximum adsorption capacity of 201.14 mg·g^−1^, close to the experimental maximum of 189.35 mg·g^−1^. Kinetic fitting and surface analyses suggest that the enhanced adsorption originates from the combined contribution of K^+^/Sr^2+^ ion exchange and coordination with oxygen-containing sites.

K_2_SbPO_6_@PGs showed good resistance to monovalent coexisting ions, while divalent Ca^2+^ and Mg^2+^ caused stronger competitive effects. After five adsorption–desorption cycles, the composite retained more than 82% of its initial adsorption capacity. Overall, the synergy between the porous PG framework and K_2_SbPO_6_ selective exchange sites provides an effective strategy for designing shaped composite adsorbents for Sr(II) removal from acidic and complex aqueous systems.

## Figures and Tables

**Table 1:** Chemical composition of MK (wt.%).

Component	CaO	SiO_2_	Al_2_O_3_	TiO_2_	MgO	Na_2_O
Content	0.084	45.788	51.687	1.276	0.173	0.116

**Table 2:** BET-derived textural parameters of PGs and K_2_SbPO_6_@PGs.

Sample	Specific Surface Area (m^2^·g^−1^)	Pore Volume (cm^3^·g^−1^)	Pore Diameter (nm)
K_2_SbPO_6_@PGs	3.144	0.014	13.18
PGs	23.924	0.131	22.20

**Table 3:** Isotherm model parameters for Sr^2+^ adsorption on PGs and K_2_SbPO_6_@PGs.

Sample	Langmuir			Freundlich			Sips			
	q_m_ (mg·g^−1^)	k_l_ (L·mg^−1^)	R^2^	*n*	k_f_ (L·mg^−1^)	R^2^	q_m_ (mg·g^−1^)	k_s_ (L·mg^−1^)	β	R^2^
PGs	84.67	0.011	0.957	34.96	0.129	0.844	74.69	0.021	1.16	0.943
K_2_SbPO_6_@PGs	225.83	0.007	0.970	27.46	0.294	0.847	201.14	0.009	1.33	0.982

**Table 4:** Comparison of Sr(II) adsorption performance of K_2_SbPO_6_@PGs with representative adsorbents reported in the literature.

Adsorbent	Material Form	Main Adsorption Mechanism	Maximum Sr(II) Adsorption Capacity	Reference
K_2_SbPO_6_@PGs	Granular porous composite	K^+^/Sr^2+^ ion exchange and oxygen-site coordination	201.14 mg·g^−1^ theoretical; 189.35 mg·g^−1^ experimental	This work
1D-K_2_SbPO_6_	Powder	K^+^/Sr^2+^ ion exchange	175.90 mg·g^−1^	[9]
K_2_SbPO_6_/PAN composite microspheres	Composite microspheres	K^+^/Sr^2+^ ion exchange	131.15 mg·g^−1^	[10]
Na-A zeolite synthesized from kaolin	Zeolite powder	Ion exchange	1.90–2.42 mmol·g^−1^, approximately 166–212 mg·g^−1^	[5]
Na_3_FePO_4_CO_3_	Crystalline phosphate/carbonate adsorbent	Na^+^/Sr^2+^ exchange and Sr-O coordination	99.6 mg·g^−1^	[4]
Layered zirconium phosphate fluoride	Layered crystalline ion exchanger	Interlayer ion exchange	161.48 mg·g^−1^	[27]
KInSnS_4_@collagen fiber aerogel	Shaped aerogel composite	K^+^/Sr^2+^ ion exchange	41.48 mg·g^−1^	[28]
LTO-MX/PAN beads	Polymer-immobilized MXene-based beads	Encapsulation, electrostatic attraction, cation exchange, and surface complexation	24.05 mg·g^−1^	[29]
Hierarchically porous zeolite–geopolymer composite	Porous geopolymer-based composite	Zeolite ion exchange and hierarchical pore-assisted mass transfer	Not directly comparable	[19]
K^+^-intercalated hierarchical titanate nanostructures	Layered titanate nanostructure	K^+^/H^+^ exchange and Sr-O interaction	204 mg·g^−1^	[30]
ZrP@magnetic graphene aerogel microspheres	Magnetic aerogel microspheres	Ion exchange and surface complexation	333.45 mg·g^−1^	[31]
Mg-MCM-41/talc composite	Mesoporous/layered hybrid adsorbent	Mainly surface interaction and physical/chemical adsorption	229.9 mg·g^−1^	[32]

## Data Availability

The original contributions presented in this study are included in the article. Further inquiries can be directed to the corresponding author.
